# Physical activity levels and self-perception among patients living with chronic conditions in France: A population-based cross-sectional study using the ComPaRe cohort

**DOI:** 10.1080/13814788.2025.2566110

**Published:** 2025-11-24

**Authors:** Thibault Triconnet, Viet-Thi Tran, Isabelle Pane, Stéphanie Sidorkiewicz

**Affiliations:** a CRESS, INSERM, INRA, Université de Paris Cité, Paris, France; b Département de médecine générale, Université de Paris Cité, Paris, France; c Centre d’Épidémiologie Clinique, Hôpital Hôtel-Dieu, AP-HP, Paris, France

**Keywords:** Physical activity, behaviour change, chronic disease, primary health care

## Abstract

**Background:**

Despite the numerous health benefits associated with physical activity (PA), many patients with chronic conditions remain inactive. We hypothesise that patients often misperceive their PA level, which affects behaviour change. We aimed to assess PA levels of patients with chronic conditions using the Global Physical activity Questionnaire (GPAQ) and compare their perception of meeting WHO guidelines (150 min of moderate-to-vigorous physical activity per week) with GPAQ measurements.

**Methods:**

We conducted a cross-sectional analysis of the GPAQ in a sample of participants recruited from the ComPaRe e-cohort, a nationwide cohort of adult patients with chronic conditions in France. We used stratified random sampling (based on age, gender and diploma) and non-responder weighting to obtain estimates representative of PA levels of chronic patients in France. Concordance between participants’ perception of meeting WHO guidelines and GPAQ measurements was assessed using Cohen’s kappa coefficient.

**Results:**

We included 629 patients (participation rate: 65.0%). The median age was 57 [46.0–65.4] years, with 348 (55.3%) women. A total of 369 (64.2%) patients were categorised as active (>750 metabolic equivalent tasks (Mets)/week) according to the GPAQ, with PA levels increasing with age among men. A total of 55 (8.6%) participants were unable to estimate their PA level, and 186 (32.4%) misperceived their PA level (cohen’s kappa coefficient of 0.38 [0.31–0.45]), with 29 (5.1%) overestimating and 157 (27.4%) underestimating their activity.

**Conclusion:**

Healthcare professionals should consider accurate screening for inactivity and patients’ self-perception of their PA level, as both are key to delivering personalised and impactful counselling.

## KEY MESSAGES

Concordance between chronically ill patients’ perceived adherence to 150 minutes of moderate-to-vigorous physical activity (PA) weekly and measurements by the Global PA Questionnaire was fair to moderate.

Approximately half of the participants were categorised as active, with PA increasing with age in men.

These findings call for rethinking PA assessment during consultations.

## Introduction

Physical activity (PA) stands as one of the major modifiable risk factors for non-communicable diseases, offering dose-dependent benefits ranging from improved mental health to reduced mortality and better control of chronic diseases [1]. The WHO launched its global action plan on PA 2018–2030 urging countries to adopt supportive national policies [2]. Several countries have implemented the recommendations of 150 min of moderate to vigorous physical activity per week into their guidelines [3]. However, despite these guidelines, only 61.5% of European adults met the PA recommendations in 2015 [4]. The situation worsened during the Covid-19 pandemic, particularly during lockdown and recovery periods [5], with limited available data on potential lifestyle changes and physical activity levels, especially among patients with chronic conditions, in the post-pandemic era.

As front-line healthcare providers, clinicians and health professionals in primary care settings are uniquely positioned to encourage discussions about physical activity with patients [6]. However, primary care PA promotion interventions or counselling have shown limited impact thus far and are heterogeneously implemented [7] because of clinicians’ lack of time and training [8], their unfamiliarity with the guidelines [9], their personal interest and engagement in physical activity [10], or other potential underexplored reasons [11].

In our study, we chose to focus on the crucial preliminary stage required before determining the most beneficial intervention or counselling for the patient: evaluating patients’ PA levels during a GP consultation. This initial step of identifying patients’ activity levels conditions the entire promotion or counselling process. Due to time constraints, primary care clinicians often rely on patients’ self-reported PA levels by using simple and direct questions, rather than validated, often lengthy questionnaires designed more for research than care. While a single-item question could offer a reasonable estimate for screening people in time-constrained settings [12], physical activity is a complex multidimensional behaviour. This complexity introduces the potential for distortions in patients’ understanding and self-awareness [11], as well as a risk of misclassification of self-perceived PA levels. This misperception may hinder the provision of appropriate and personalised advice from the physician side, and act as a barrier to the successful application of most theoretical models of behaviour change from the patient side [13]. Notably, patients who overestimate their PA levels may be less likely to effectively change their PA behaviour. Furthermore, patients living with chronic conditions appear to be at least as vulnerable as the general population to misperceptions regarding their physical activity levels [14,15].

In the present study, we aimed to measure PA levels in the French population of patients living with chronic conditions, using the Global Physical Activity Questionnaire (GPAQ) and examine the concordance with patients’ responses to a simple question regarding their self-perception of meeting WHO guidelines.

## Methods

This study is based on a cross-sectional survey nested within the ComPaRe (Community of Patients for Research) e-cohort [16]. ComPaRe is a nationwide cohort of adult patients with at least one chronic condition (defined as a condition requiring healthcare for at least 6 months) and who voluntarily join the project to contribute to research by regularly completing online questionnaires on patient-reported outcomes and experiences, and by taking part in nested studies. Approximately 70,000 participants are enrolled in the e-cohort. The protocol was registered on the Open Science Framework before the inclusion of the first patient: https://osf.io/6cdht.

### Population

A stratified random sampling was carried out within the cohort, based on age (18–34 y/35–64y/65y and older), last diploma obtained (bachelor’s degree or less/more than bachelor’s degree) and gender (women/men) to align with the characteristics of the population of people living with chronic conditions, in France [17,18]. Random invitations were sent to patients within each of the 12 resulting strata, who had logged into their ComPaRe platform account within the last 18 months and had given consent to participate to additional external studies nested in ComPaRe. The final sample was weighted to account for non-responders. The ComPaRe cohort was approved by the ethics committee of the Hôtel-Dieu Hospital, Paris, France (IRB: 0008367). All participants provided electronic consent before participating in the ComPaRe cohort.

### Measures

#### Measurement of patients’ physical activity level by a validated questionnaire (GPAQ)

Physical activity was measured using the French version of the GPAQ [19]. The GPAQ, developed by the WHO, is now recommended for national surveillance of PA [3]. It includes 16 items assessing PA (yes/no question), frequency (number of days per week) and duration (in hours and minutes) in three domains: activity at work, travels to and from places, and recreational activities. For each domain, PA duration by minutes per day and week allows the calculation of energy expenditure, scored in metabolic equivalent tasks (METs). The French validated version demonstrated acceptable validity and reliability for PA measurement (ICC = 0.37–0.94; *κ* = 0.50–0.62; *r* = 0.41–0.86) [19,20].

#### Measurement of patients’ physical activity level by a single unidimensional question about self-perception of meeting WHO guidelines

Perception of reaching the WHO recommended threshold was collected using a single question with three response options: “*During a typical week, including weekends: 1) you engage in 150 min or more (2.5 h) of moderate to vigorous physical activity; 2) you engage in less than 150 min (2.5 h) of moderate to vigorous physical activity; 3) Don’t know*.” This question was designed to model a classic assessment by a GP during consultation. Before the question, moderate to vigorous physical activity was defined as physical activity that requires a moderate amount of effort, noticeably accelerates the heart rate, and leads to faster breathing and feeling warmer [21]. Participants were asked to answer this question before completing the GPAQ. All our data were collected between April and July 2022.

#### Other variables

Socio-demographic characteristics (e.g. age, sex), number and chronic conditions using the International Classification of Primary Care-Version 2 were obtained from data already collected in the ComPaRe cohort. The survey also collected participants’ temporary or permanent limitation to PA, teleworking status, and the use of wearable biometric monitoring devices (for monitoring PA or for other purposes). Additionally, it collected whether health care professionals pointed out patients’ lack of PA and/or provided advice for PA during the past year.

The survey underwent pilot testing and review with 6 patients of various genders, ages, and professional characteristics to enhance clarity, understanding, and completion duration.

### Statistical analysis

Descriptive statistics of the sample and levels of PA, according to the GPAQ, were calculated.

GPAQ measurements were corrected by age, BMI and gender using to the method proposed by Kozey et al. [22] and dichotomised into ≥750 METs/week vs. <750 METs/week to determine whether participants had PA levels over or under the threshold recommended by the WHO [3]. Patients who reported engaging in more than 150 min per week were classified as active, those who reported engaging in less were classified as inactive. We also conducted two post hoc sensitivity analyses using the respective thresholds of 600 METs and 900 METs, which are occasionally found in the literature (available in Supplementary Appendix 1).

Agreement between perceived levels of reaching the WHO guidelines and measurements from the GPAQ was assessed by determining the number and percentage of “over-estimators”, “under-estimators”, “realistically active” and “realistically inactive” [11] and by calculating Cohen’s Kappa coefficient (ĸ) and its 95% confidence interval [23]. The kappa coefficient was interpreted as [24]: 1–0.81 almost perfect, 0.80 − 0.61 substantial, 0.60–0.41 moderate, 0.40–0.21 fair, 0.20–0.00 slight and <0 poor.

The sample size was determined based on the agreement between perceived levels of PA and measurements from the GPAQ. Assuming an agreement of *ĸ* = 0.55 according to data from Godino et al. [25], a probability of meeting the threshold from the WHO of 61% [4,26], a sample size of 502 patients was required to obtain a precision of ±0.075. Considering a response rate of 50%, we estimated that a total of 1004 participants were to be invited.

All analyses have been performed with R version 4.2.1 (http://www.r-project.org). The STROBE guidelines were adhered to for reporting the results.

## Results

### Patient characteristics

A total of 972 eligible patients were contacted by mail, and 629 completed the questionnaire (15 refused to participate, 19 did not fully complete the questionnaire, 5 had died prior to the invitation; participation rate 65%). The participation rate in each stratum is available in Supplementary Appendix 2.

After weighting for non-responders, median age was 57, interquartile range (IQR) 46.0 to 65.4 years, and 55.3% were women. Employed and students accounted for 238 (37.9%), while retired people were 251 (40%). Our study population had a median of 2.5 [1.0;4.0] chronic conditions, with the most prevalent being high blood pressure (16.9%), chronic low back pain (13%) and diabetes (11.3%). All the patient characteristics are detailed in Table 1 and the list of chronic conditions reported by the patients is provided in Supplementary Appendix 4.

**Table 1.** Characteristics of the study population (*n* = 629).

Characteristics	Raw data	Weighted data^b^
Age—median [IQR], years	61 [45.0–69.0]	57 [46.0–65.4]
Female sex—*n* (%)	334 (53.0)	348 (55.3)
BMI—median [IQR]	25.7 [22.6–29.1]	25.3 [22.0–29.6]
Size of the residential area—*n* (%)		
<2.000 inhabitants	121 (19.2)	121 (19.3)
2.000 to 50.000 inhabitants	294 (46.7)	294 (46.8)
>50.000 inhabitants	214 (34.0)	213 (33.9)
Level of education—*n* (%)		
No degree	29 (4.6)	29 (4.7)
Middle school degree equivalent	61 (9.4)	63 (9.9)
Technical institutes degree	164 (26.1)	166 (26.3)
Bachelor’s degree or equivalent diploma	228 (36.2)	234 (37.2)
2 years post bachelor diploma or certificate	41 (6.5)	37 (5.6)
5 years post bachelor diploma or certificate or more	106 (16.9)	99 (15.8)
Working status—*n* (%)		
Having a job / employed	213 (33.9)	214 (34.1)
Out of the labour force (including unemployed, staying-at-home indivuals, disabled)	124 (19.7)	126 (20.0)
In training or student	20 (3.2)	24 (3.8)
Retired	259 (41.2)	251 (40.0)
Other	13 (2.1)	13 (2.1)
Economic status—*n* (%)		
“I need to go into debt to manage”	15 (2.4)	15 (2.5)
“It is difficult”	60 (9.5)	61 (9.8)
“A bit tight, I must watch my finances”	130 (20.7)	131 (20.8)
“At equilibrium”	177 (28.1)	178 (28.3)
“Rather comfortable”	129 (20.5)	124 (19.7)
“Very comfortable”	27 (4.3)	26 (4.1)
Chronic diseases^a^—*n* (%)		
High blood pressure	122 (19.4)	106 (16.9)
Chronic low-back pain	94 (14.9)	82 (13.0)
Diabetes	82 (13.0)	71 (11.3)
Long COVID	67 (10.7)	58 (9.2)
Endometriosis	55 (8.7)	48 (7.6)
Depression	46 (7.3)	40 (6.4)
Asthma	44 (7.0)	38 (6.1)
Osteoarthrosis	42 (6.7)	37 (5.8)
Dyslipidemia	40 (6.4)	35 (5.5)
Sleep apnoea syndrom	38 (6.0)	33 (5.3)

^a^For sake of clarity, we choose to report here only the ten most prevalent diseases. All the results are detailed in Supplementary Appendix 4. ^b^Weighted data on non-responders (age, diploma, gender).

Abbreviations: IQR: Interquartile range.

Among participants, 57.6% had a connected device, of which 21.2% never consulted the PA data (pedometer or other), 9.1% consulted them about once a month, 17.4% consulted them about once a week and 52.2% consulted them almost every day.

Regarding discussions about PA during consultations, 323 (51.4%) of the study participants reported having received PA recommendations from a physician, of which 82.0% were general and 18.0% were specific ones (including details such as the type or intensity of activity, along with duration and/or frequency). Among those who received recommendations, 29.3% indicated that their doctor took their lifestyle into account in counselling them.

### Measurement of PA levels using the GPAQ

**Figure 1.**
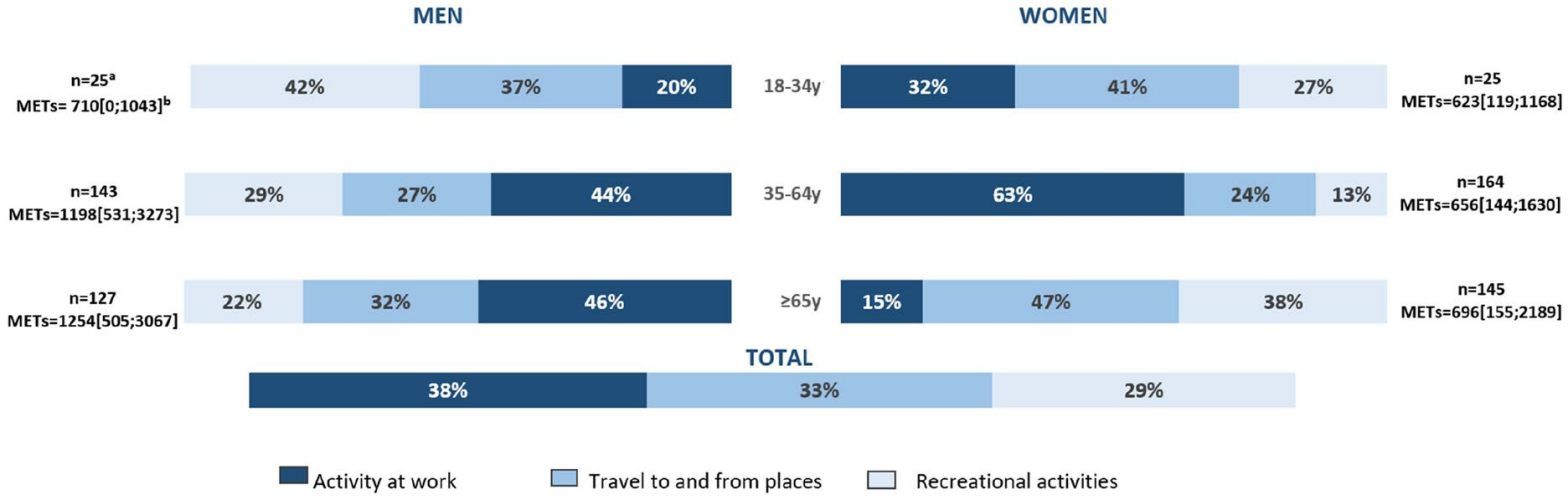
Distribution of participants’ weekly physical activity according to age and gender (*n* = 629). ^a^Population by group after weighting on non-responders. ^b^Median METs/week and IQR after correction by the gender, age, BMI as described by Kozey et al. [21] and weighting on non-responders.

**Figure 2.**
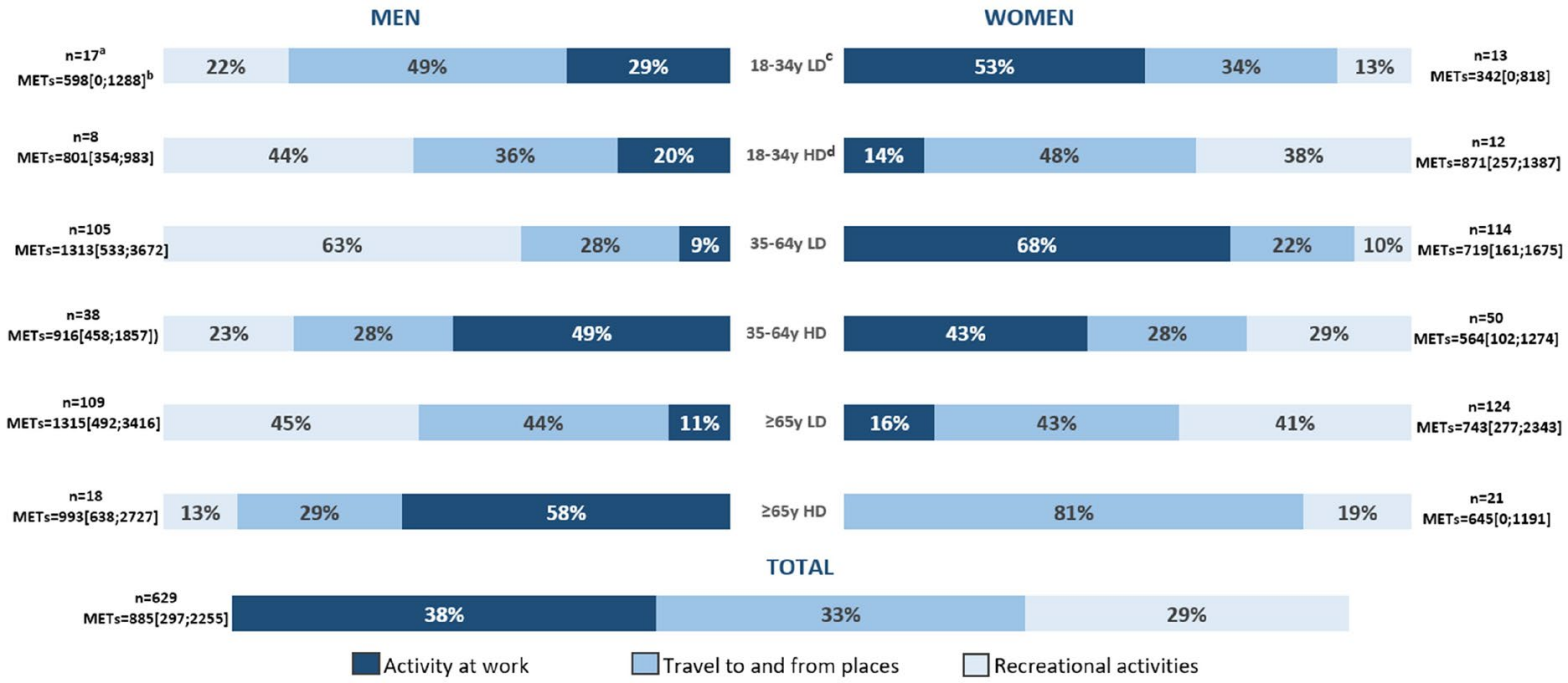
Distribution of participants’ weekly physical activity according to age, gender and last diploma obtained (*n* = 629). ^a^Population by group after weighting on non-responders. ^b^Median METs/week and IQR after correction by the gender, age, BMI as described by Kozey et al. [21] and weighting on non-responders. ^c^LD stands for Lower Diploma: high school diploma and below. ^d^HD stands for Higher Diploma: above high school diploma.

A total of 314 (54.7%) patients were categorised as active (>750METs/week) based on the GPAQ. The median number of METs/week was 885 [297;2255]. Activity at work constituted 38% of the weekly total physical activity volume, while travel to and from places accounted for 33% and recreational activities for 29%. Among all participants, 23.2% teleworked, and, on average, 3 days a week. The distribution of participants’ weekly PA categorised into the three domains of the GPAQ and according to age and gender are illustrated in Figure 1. The amount of PA at work varies greatly based on the level of last diploma obtained. For example, among the participants aged 65 years or older, the percentage of physical activity at work was 11 and 16%, respectively, for men and women with lower diploma, compared to 58% and 0%, respectively, for men and women with higher diploma. The distribution of participants’ weekly PA categorised into the three domains and according to age, gender and diploma are illustrated in Figure 2.

### Patients’ self-perception of meeting WHO guidelines

Regarding patients’ perception of meeting WHO guidelines, 241 (42.0%) considered themselves as meeting the WHO guidelines, while 333 (58.0%) did not; 55 (8,6%) were unable to estimate their PA level.

In comparison with the GPAQ assessment, 192 (33.5%) patients correctly identified themselves as active, and 211 (36.8%) patients correctly identified themselves as inactive. On the other hand, 49 (8.5%) patients overestimated their level of physical activity, and 122 (21.3%) patients underestimated their level of physical activity, totalling 171 (29.8%) patients who misperceived their PA level.

Regarding the concordance between patients’ perception of meeting the guideline threshold and the measurement by the GPAQ, we excluded the 55 patients who answered “I don’t know” to the question exploring their perception and analysed a total of 574 answers (Table 2). Cohen’s kappa coefficient was 0.41 [0.34–0.48], indicating a fair-to-moderate level of agreement [24]. Before correction and weighting on non-responders, the raw Cohen’s kappa coefficient was 0.43 [0.36–0.50] (raw data are available in Supplementary Appendix 3).

**Table 2.** Concordance between chronic patients’ perception of reaching WHO guidelines on physical activity and the measurement of patients’ physical activity by the GPAQ (*n* = 574).

		Measurement of physical activity by the GPAQ	
		Inactive *n* = 260 (45.3%)	Active *n* = 314 (54.7%)
Self-perception of reaching 150 min of moderate to vigorous physical activity^b^	Inactive *n* = 333 (58.0%)	Realistically inactive *n* = 211 (36.8%)	Underestimator *n* = 122 (21.3%)
	Active *n* = 241 (42.0%)	Overestimator *n* = 49 (8.5%)	Realistically active *n* = 192 (33.5%)
Concordance			
Kappa Cohen corrected^a^		*K* = 0.41 [0.34–0.48]	

^a^After correction of the MET measure in GPAQ by the gender, age, BMI as described by Kozey et al. [21] and weighting on non-responders.^b^55(8.6%) patients responded “I don’t know” to the perception question and therefore were not included in this analysis.

## Discussion

### Summary

Within the chronically ill population in France, the prevalence of meeting PA recommendations following three years of the COVID-19 pandemic is 54.7%. We found that nearly 29.8% of patients presenting at least one chronic condition do not correctly estimate their level of physical activity when responding to a simple unidimensional question and that 8.6% are not able to estimate it. Our study is the first, to our knowledge, to assess the concordance between patients’ perception of reaching WHO guidelines on physical activity and the measurement of their PA by a validated tool in a representative sample of patients with chronic conditions in France.

### Comparison with existing literature

The prevalence results regarding adherence to physical activity recommendations are consistent with other European studies conducted in populations with chronic diseases [27–29]. It seems that patients with chronic diseases are more accurate in estimating their level of physical activity than the general population, with 64% of patients estimating themselves correctly, whereas other studies have reported figures around 50% [14,15]. We also observed a higher proportion of under-estimators than over-estimators. This latter result might be explained—in addition to statistical fluctuation – by the nature of our study population. Patients with chronic conditions may have had a greater tendency to underestimate their physical activity level than the general population, supporting the hypothesis that people in better health have a greater tendency to overestimate their PA level [11,14]. The validity of physical activity measures may also differ depending on the degree of fitness of individuals [30].

### Strengths and limitations

Our study has several limitations. The first limitation is a selection bias related to the ComPaRe cohort, including patients who have access to the Internet and voluntarily agreed to participate in research studies. They are more committed to their health and are more accustomed to answering self-questionnaires measuring various health dimensions. We tried to minimise this selection bias through stratified random sampling, leading to the construction of a representative sample of the French chronically ill population in terms of age, level of study and gender. We also have made all possible efforts to minimise the non-response rate, resulting in a good participation rate, compared to other studies with similar designs and data collection [14,31]. The second limitation is the use of self-administered questionnaires, at risk of classification bias [32]. We attempted to minimise this bias by using the GPAQ, a validated and widely used tool [33] and by correcting the measurement of METs [22] to improve its accuracy. The use of more objective measures as three-dimensional accelerometers was not feasible to implement in the ComPaRe cohort due to logistical constraints, but should be considered in future studies. The third limitation is the use of a non-validated item to assess the self-perception of patients in reaching WHO guidelines’ threshold. However, while several single-item measurements have been evaluated in the literature [12], no validated question in French specifically addressed the 150-minute weekly volume, which aligns with the updated WHO guidelines and accounts for individuals whose activity levels may fluctuate across different days.

The main strength of this study lies in its reliance on a large and representative population of patients with chronic conditions, in contrast to previous studies more focused on subgroups of population. Additionally, the use of the GPAQ allowed us to provide a three-dimensional description of patients’ physical activity. Finally, leveraging the ComPaRe e-cohort enabled the collection patients’ self-reports through online questionnaires rather than face-to-face encounters potentially reducing the influence of social desirability bias, but this may limit comparisons with studies carried out in other data collection contexts.

### Clinical implications

The promotion of PA remains a major public health challenge [2] given that PA has been described as the “miracle cure” [6]. Currently, what seems to be the most effective techniques for engaging patients in PA in long term are personalised prescriptions including the use of feedback, goal setting, and shared decision making [34–36], with PA promotion interventions that take complexity of mechanisms involved in behaviour change into account [37]. However, the success of these strategies relies on the accuracy of patients’ self-assessments. Willingness to change is strongly related to the perception of the behaviour to be changed and to the self-assessment of this behaviour [15]. Our study suggests that healthcare providers should consider the possibility of misestimation and use more objective tools when appropriate. Wearable technologies such as connected watches and smartphones offer promising opportunities, with an increasing number of studies evaluating the validity and reliability of the measurements they provide [38]. Another possible approach could involve the use of validated questionnaires completed outside the consultation time. Furthermore, in a broader context of trying to achieve minimally disruptive medicine for patients with chronic conditions [39], this study provides valuable preliminary insights for tailoring PA promotion interventions. Differentiating between over-estimators—who may be less receptive to behavioural advice—and under-estimators—who may experience undue burden or discouragement—could allow for more tailored, effective physical activity promotion strategies. This aligns with a shift towards more holistic, patient-centered approaches in healthcare.

### Research implications

Future research should explore innovative methods for accurately screening for inactivity in the primary care context. This may involve expanding beyond the current model of constrained short patient-GP consultations and viewing the patient’s care journey as a continuous process. This approach would enable all healthcare professionals to seise every opportunity (particularly in preparation for a consultation or between two consultations, and no longer necessarily and only during the traditional patient-GP encounter) for a more accurate assessment of the patient’s PA level using the method best suited to their preferences and individual characteristics. This approach could involve the use of digital tools or pre-visit questionnaires, and the help of nurse practitioners, or medical assistants. There is also a need for more high-quality, evidence-based studies focusing on counselling for lifestyle changes, along with broader reflection on the development of community-based interventions [40].

## Conclusions

Our study reveals moderate concordance between patients’ perceived adherence to WHO physical activity guidelines and measurements obtained using the validated Global Physical Activity Questionnaire (GPAQ) within a representative sample of chronically ill patients in France. The importance of accurate screening for inactivity and patients’ moderate self-perception of their PA level should be considered by healthcare professionals to facilitate personalised PA counselling.

## Supplementary Material

Supplemental Material

## Patient and public involvement

Patients and/or the public were not involved in the design, or conduct, or reporting, or dissemination plans of this research.

## Data abailability statement

Data are available on reasonable request.
