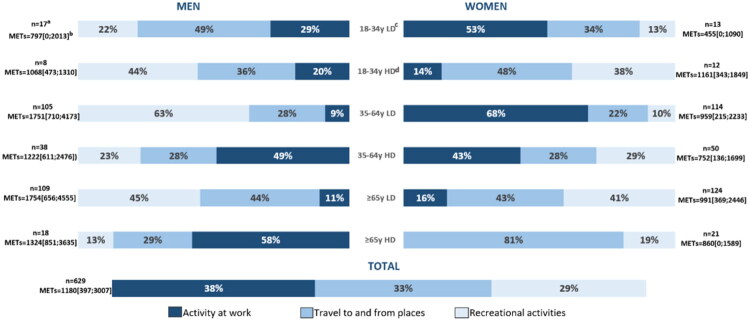# Correction

**DOI:** 10.1080/13814788.2026.2620941

**Published:** 2026-02-02

**Article title:** Physical activity levels and self-perception among patients living with chronic conditions in France: A population-based cross-sectional study using the ComPaRe cohort

**Authors:** Thibault Triconnet, Viet-Thi Tran, Isabelle Pane, Stephanie Sidorkiewicz

**Journal:** *IGEN: European Journal of General Practice*

**DOI:** https://doi.org/10.1080/13814788.2025.2566110

Several sections of text were published incorrectly. Below are the original text and the updated text:

**Original**:

A total of 314 (54.7%) patients were categorised as active (>750METs/week) based on the GPAQ. The median number of METs/week was 885 [297;2255].

**Updated**:

A total of 369 (64.2%) patients were categorised as active (>750METs/week) based on the GPAQ. The median number of METs/week was 1180 [397;2792].

**Original**:

Regarding patients’ perception of meeting WHO guidelines, 241 (42.0%) considered themselves as meeting the WHO guidelines, while 333 (58.0%) did not; 55 (8,6%) were unable to estimate their PA level. In comparison with the GPAQ assessment, 192 (33.5%) patients correctly identified themselves as active, and 211 (36.8%) patients correctly identified themselves as inactive. On the other hand, 49 (8.5%) patients overestimated their level of physical activity, and 122 (21.3%) patients underestimated their level of physical activity, totalling 171 (29.8%) patients who misperceived their PA level. Regarding the concordance between patients’ perception of meeting the guideline threshold and the measurement by the GPAQ, we excluded the 55 patients who answered “I don’t know” to the question exploring their perception and analysed a total of 574 answers (Table 2). Cohen’s kappa coefficient was 0.41 [0.34–0.48], indicating a fair-to-moderate level of agreement [24]. Before correction and weighting on non-responders, the raw Cohen’s kappa coefficient was 0.43 [0.36–0.50] (raw data are available in Supplementary Appendix 3).

**Updated**:

In comparison with the GPAQ assessment, 212 (36.9%) patients correctly identified themselves as active, and 176 (30.6%) patients correctly identified themselves as inactive. On the other hand, 29 (5.1%) patients overestimated their level of physical activity, and 157 (27.4%) patients underestimated their level of physical activity, totalling 186 (32.4%) patients who misperceived their PA level. Regarding the concordance between patients’ perception of meeting the guideline threshold and the measurement by the GPAQ, we excluded the 55 patients who answered “I don’t know” to the question exploring their perception and analysed a total of 574 answers (Table 2). Cohen’s kappa coefficient was 0.38 [0.31-0.45], indicating a fair-to-moderate level of agreement [24]. Before correction and weighting on non-responders, the raw Cohen’s kappa coefficient was 0.42 [0.35-0.49] (raw data are available in Supplementary Appendix 3).

**Original**:

Within the chronically ill population in France, the prevalence of meeting PA recommendations following three years of the COVID-19 pandemic is 54.7%. We found that nearly 29.8% of patients presenting at least one chronic condition do not correctly estimate their level of physical activity…

**Updated**:

Within the chronically ill population in France, the prevalence of meeting PA recommendations following three years of the COVID-19 pandemic is 64.2%. We found that nearly 32.4% of patients presenting at least one chronic condition do not correctly estimate their level of physical activity…

**Original**:

It seems that patients with chronic diseases are more accurate in estimating their level of physical activity than the general population, with 64% of patients estimating themselves correctly…

**Updated**:

It seems that patients with chronic diseases are more accurate in estimating their level of physical activity than the general population, with 62% of patients estimating themselves correctly…

These have now been corrected.

The Table 2 data figures were originally submitted incorrectly. This has now been corrected:

**Original**:

**Table:** 

		Measurement of physical activity by the GPAQ	
		Inactive n = 260 (45.3%)	Active n = 314 (54.7%)
Self-perception of reaching 150 minutes of moderate to vigorous physical activity^b^	Inactive n = 333 (58.0%)	Realistically inactive n = 211 (36.8%)	**Underestimator** **n = 122 (21.3%)**
	Active n = 241 (42.0%)	**Overestimator** **n = 49 (8.5%)**	Realistically active n = 192 (33.5%)
Concordance			
Kappa Cohen corrected^a^		K = 0.41 [0.34-0.48]	

**Updated**:

**Table:** 

		Measurement of physical activity by the GPAQ	
		Inactive n= 205 (35.7%)	Active n = 369 (64.2%)
Self-perception of reaching 150 minutes of moderate to vigorous physical activity^b^	Inactive n = 333 (58.0%)	Realistically inactive n = 176 (30.6%)	**Underestimator** **n = 157 (27.4%)**
	Active n = 241 (42.0%)	**Overestimator** **n = 29 (5.1%)**	Realistically active n = 212 (36.9%)
Concordance			
Kappa Cohen corrected^a^		K = 0.38 [0.31-0.45]	

The originally published Figures 1 & 2 contained errors in some of the data points. They have now been corrected.

**Original Figure 1:**


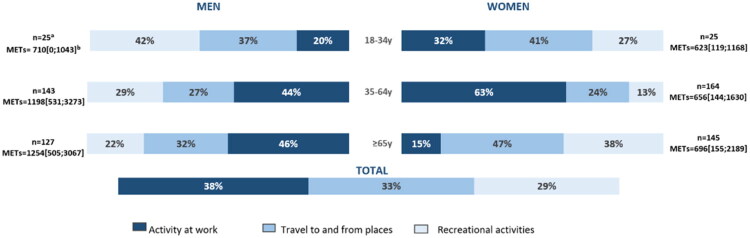


**Updated Figure 1:**


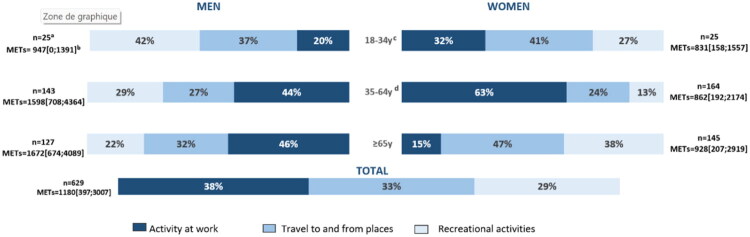


**Original Figure 2:**


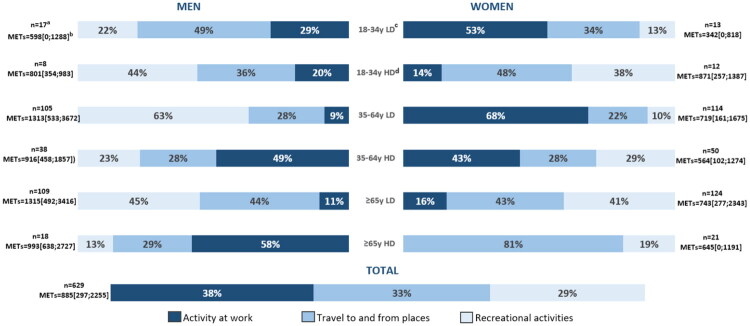


**Updated Figure 2:**