# Supplementary material for: Physical activity levels and self-perception among patients living with chronic conditions in France: A population-based cross-sectional study using the ComPaRe cohort
Source: Eur J Gen Pract. 2025 Nov 24;31(1):2566110. doi: 10.1080/13814788.2025.2566110 (PMC12646102; doi:10.1080/13814788.2025.2566110)
Supplement: Supplemental Material [file IGEN_A_2566110_SM7350.docx]

**SUPPLEMENTAL MATERIALS**

**Supplemental appendix 1. Sensitivity analysis**

**With METs Threshold fixed to 600**

**Concordance between chronic patients' perception of reaching WHO guidelines on physical activity and the measurement of patients’ physical activity by the GPAQ (n=574)**

|  | | **Measurement of physical activity by the GPAQ** | |
| --- | --- | --- | --- |
|  |  | **Inactive n= 182(31.7%)** | **Active n= 392(68.3%)** |
| **Self-perception of reaching 150 minutes of moderate to vigorous physical activity** | **Inactive n=333(58.0%)** | **Realistically inactive**  **n= 159(27.7%)** | **Underestimator**  **n= 174(30.3%)** |
|  | **Active n=241(42.0%)** | **Overestimator**  **n= 23(4.0%)** | **Realistically active**  **n=218(38.0%)** |
| **Concordance** | | | |
| **Kappa Cohen corrected** | | **K=0.35 [0.29-0.41]** | |

**With METs Threshold fixed to 900**

**Concordance between chronic patients' perception of reaching WHO guidelines on physical activity and the measurement of patients’ physical activity by the GPAQ (n=574)**

|  | | **Measurement of physical activity by the GPAQ** | |
| --- | --- | --- | --- |
|  |  | **Inactive n=239(41.6%)** | **Active n=335(58.4%%)** |
| **Self-perception of reaching 150 minutes of moderate to vigorous physical activity** | **Inactive n=333(58.0%)** | **Realistically inactive**  **n= 200(34.8%)** | **Underestimator**  **n= 132(23.0%)** |
|  | **Active n=241(42.0%)** | **Overestimator**  **n= 38(6.6%)** | **Realistically active**  **n= 203(35.3%)** |
| **Concordance** | | | |
| **Kappa Cohen corrected** | | **K=0.42 [0.35-0.49]** | |

**Supplemental Appendix 2. Participation rate in each stratum**

| **Strata** | **Participation rate (%)** |
| --- | --- |
| Men 18-34yo |  |
| *Bachelor or less* | 62,8 |
| *More than Bachelor* | 61,7 |
| Men 35-64yo |  |
| *Bachelor or less* | 63,6 |
| *More than Bachelor* | 66 |
| Men >65yo |  |
| *Bachelor or less* | 75,3 |
| *More than Bachelor* | 74,7 |
| Female 18-34yo |  |
| *Bachelor or less* | 49,4 |
| *More than Bachelor* | 58,8 |
| Female 35-64yo |  |
| *Bachelor or less* | 63,4 |
| *More than Bachelor* | 69,2 |
| Female >65yo |  |
| *Bachelor or less* | 57,9 |
| *More than Bachelor* | 92 |
| Total | 65 |

**Supplemental Appendix 3. Raw concordance data.**

**Concordance between chronic patients' perception of reaching WHO guidelines on physical activity and the measurement of patients’ physical activity by the GPAQ (n=575)**

|  | | **Measurement of physical activity by the GPAQ** | |
| --- | --- | --- | --- |
|  |  | Inactive n=237(41.3%) | Active n=338(58.9%) |
| **Self-perception of reaching 150 minutes of moderate to vigorous physical activity^b^** | Inactive n=331(57.6%) | Realistically inactive  n=199(34.7%) | Underestimator  n=132(22.3%) |
|  | Active n=244(42.4%) | Overestimator  n=38(6.62%) | Realistically active  n=206(35.9%) |
| **Concordance** | | | |
| Kappa Cohen raw | | K=0.42[0.35-0.49] | |
| Kappa Cohen corrected^a^ | | K=0.38 [0.31-0.45] | |

^a^After correction of the MET measure in GPAQ by the gender, age, bmi as described by Kozey et al.(34) and weighting on non-responders.^b^ 54(8.6%) patients responded “I don’t know to the perception question and therefore were not included in this analysis.

**Supplemental Appendix 4. List of chronic conditions reported by patients.**

| **Chronic condition** | **Occurrence** |
| --- | --- |
| High blood pressure | 122 |
| Chronic low back pain | 94 |
| Diabetes | 82 |
| Long Covid | 67 |
| Endometriosis | 55 |
| Depression | 46 |
| Asthma | 44 |
| Arthrosis | 42 |
| Dyslipidemia | 40 |
| Sleep Apnea | 38 |
| Fibromyalgia | 34 |
| COPD and emphysema | 28 |
| Vitiligo | 28 |
| Chronic renal insufficiency | 27 |
| Neurofibromatosis type 1 | 27 |
| Gastroesophageal reflux, achalasia, hiatal hernia | 27 |
| Axial spondyloarthritis | 25 |
| Hypothyroidia | 24 |
| Chronic Cephalea (including migraine) | 23 |
| Rheumatoid arthritis | 21 |
| Psoriasis | 21 |
| Osteoporosis / Osteopenia | 20 |
| Chronic cervical pain, chronic back pain | 19 |
| Peripheral neuropathy | 19 |
| Anxiety disorder | 19 |
| Scoliosis | 18 |
| Hashimoto thyroidis | 18 |
| Irritable bowel syndrome / Functional colopathy | 17 |
| Vision problems (myopia, presbyopia, astigmatism) | 17 |
| History of myocardial infarction | 15 |
| Benign hypertrophy (adenoma) of the prostate | 15 |
| Allergy | 14 |
| Verneuil's disease / hidradenitis suppurativa | 14 |
| Obesity (Body Mass Index>30) | 14 |
| Acute Covid-19 (WITHOUT persistent symptoms) | 13 |
| HIV infection, AIDS | 13 |
| Atrial fibrillation cardiac arrhythmia: ACFA | 11 |
| Prostate cancer | 11 |
| Marfan syndrome | 11 |
| Chronic tendonitis (periarthritis, epicondylitis, capsulitis...) | 11 |
| Other cardiac or vascular disease | 10 |
| Breast cancer | 10 |
| Chronic glaucoma | 10 |
| Bipolar disorder, manic-depressive illness | 10 |
| Heart rhythm disorders (including Bouveret's disease) | 10 |
| Crohn's disease | 9 |
| Multiple sclerosis plaque | 9 |
| Adenomyosis | 8 |
| Angina/angina pectoris/coronary artery disease/coronary heart disease (without myocardial infarction) | 8 |
| Other renal, urinary or genital disease | 8 |
| Insipid diabetes | 8 |
| Epilepsy | 8 |
| Urinary incontinence | 8 |
| Parkinson's disease | 8 |
| Psoriatic arthritis | 8 |
| Goujerot-Sjogren syndrome | 8 |
| History of phlebitis or pulmonary embolism | 7 |
| Other rheumatologic disease | 7 |
| Diverticula | 7 |
| Eczema (atopic dermatitis) | 7 |
| Heart failure | 7 |
| Chronic rhinitis | 7 |
| Chronic sinusitis | 7 |
| Ehlers Danlos syndrome | 7 |
| Chronic fatigue syndrome (Myalgic encephalomyelitis) | 7 |
| Restless legs syndrome | 7 |
| Complex regional pain syndrome (CRPS 1 or algodystrophy and CRPS 2 or algoneurodystrophy) | 7 |
| Urticaria | 7 |
| Facial Vascular Algebra | 6 |
| Other neurological disease | 6 |
| ENT cancer (lip, mouth, pharynx, larynx...) | 6 |
| Hepatitis B | 6 |
| Graves' disease | 6 |
| Myasthenia | 6 |
| Polycystic kidney disease | 6 |
| Hemorrhagic rectocolitis (UC) | 6 |
| Hearing disorders / deafness / tinnitus | 6 |
| Gastro duodenal ulcer, chronic gastritis | 6 |
| Vertigo (Meniere's disease, paroxysmal positional vertigo) | 6 |
| Cancer of the lung, bronchus, and pleura | 5 |
| Cataract | 5 |
| Hemochromatosis | 5 |
| Ovarian cyst, polycystic ovarian syndrome (PCOS) | 5 |
| Lyme disease | 5 |
| Addictions (alcohol, gambling, other drugs...) | 4 |
| Trigeminal pain (essential neuralgia, neuropathic pain of the trigeminal nerve) | 4 |
| Colon cancer | 4 |
| Kidney cancer | 4 |
| Herpes | 4 |
| Pancreatic insufficiency (chronic pancreatitis) | 4 |
| Chronic urinary lithiasis (kidney stones) | 4 |
| Raynaud's disease | 4 |
| Interstitial lung disease (idiopathic fibrosis, hypersensitivity pneumonia) | 4 |
| Scleroderma | 4 |
| Hepatic steatosis (NASH) | 4 |
| Valvular disease, valve replacement (including Barlow's disease) | 4 |
| Amyloidosis | 3 |
| History of transient ischemic attack (TIA) | 3 |
| History of cerebrovascular accident (CVA) | 3 |
| Carotid arteritis/stenosis/lower extremity arterial disease obliterans | 3 |
| Microcrystalline arthropathies (gout, chondrocalcinosis) | 3 |
| Other Hyperthyroidism | 3 |
| Other digestive system disease | 3 |
| Skin cancer of the basal cell carcinoma type | 3 |
| Thyroid cancer | 3 |
| Chronic venous insufficiency | 3 |
| Chronic lymphocytic leukemia | 3 |
| Lichen planus | 3 |
| Lupus | 3 |
| Addison's disease (adrenal insufficiency) | 3 |
| Arnold's neuralgia | 3 |
| Pelade | 3 |
| Colonic polyps | 3 |
| Chronic polyradiculonevritis | 3 |
| Chronic psychosis (schizophrenia...) | 3 |
| Immunological thrombocytopenic purpura | 3 |
| Sarcoidosis | 3 |
| Ductal syndromes (Carpal tunnel syndrome, Guyon's lodge syndrome?) | 3 |
| Personality disorder | 3 |
| Eating disorder (anorexia, bulimia?) | 3 |
| Erectile disorder | 3 |
| Shingles | 3 |
| Other cancer | 2 |
| Other eye disease | 2 |
| Other endocrine disease | 2 |
| Other ENT or respiratory disease | 2 |
| Esophageal cancer | 2 |
| Pancreatic cancer | 2 |
| Congenital heart disease | 2 |
| Retinal or vitreous detachment | 2 |
| Clotting factor deficiency/clotting disorders | 2 |
| Pulmonary arterial hypertension | 2 |
| Chronic myeloid leukemia | 2 |
| Lymphoedema | 2 |
| Celiac disease | 2 |
| Behçet's disease | 2 |
| Berger's disease | 2 |
| Charcot-Marie-Tooth disease | 2 |
| Churg and Strauss disease (eosinophilic granulomatosis with polyangiitis) | 2 |
| Gilbert's disease | 2 |
| Takayasu disease | 2 |
| Arnold-Chiari malformation | 2 |
| Mastocytosis, mast cell activation syndrome (MCAS) | 2 |
| Melanoma | 2 |
| Meningioma | 2 |
| Myeloma (monoclonal gammopathy, MGUS, plasmacytoma, Waldenstrom disease) | 2 |
| Acquired myopathy (inflammatory, dermatomyositis, polymyositis, other) | 2 |
| Neurofibromatosis type 2 (NF2) or central neurofibromatosis | 2 |
| Naso-sinusal polyposis | 2 |
| Rosacea | 2 |
| Post traumatic stress syndrome | 2 |
| Myeloproliferative syndrome (polycythemia, Vaquez disease, essential thrombocythemia, primary myelofibrosis) | 2 |
| Conduction disorder (Pace Maker) | 2 |
| Sleep disorders | 2 |
| Obsessive-compulsive disorder: OCD | 2 |
| Pituitary adenoma | 1 |
| Biermer's anemia (megaloblastic anemia, pernicious anemia) | 1 |
| Autism | 1 |
| Other skin disease | 1 |
| Other systemic disease | 1 |
| Other constitutional hemolysis (thalassemia, spherocytosis) | 1 |
| Squamous cell carcinoma of the skin | 1 |
| Bladder cancer | 1 |
| Cervical cancer of the uterus | 1 |
| Primary sclerosing cholangitis | 1 |
| Cryoglobulinemia | 1 |
| Relapsing cystitis | 1 |
| Immune deficiency (excluding HIV) | 1 |
| Macular degeneration (AMD) | 1 |
| Seborrheic dermatitis | 1 |
| Bronchial dilatation | 1 |
| Myotonic dystrophy, muscular dystrophy | 1 |
| Endocarditis | 1 |
| Alcoholic hepatitis/cirrhosis | 1 |
| Metabolic hepatitis/cirrhosis | 1 |
| Multiple chemical hypersensitivity - MCS | 1 |
| Bone and joint infection (arthritis/spondylodiscitis) | 1 |
| Tarlov cysts, peri-radicular cyst, meningeal sacral cyst | 1 |
| Non-Hodgkin's lymphoma | 1 |
| Alzheimer's disease | 1 |
| Dupuytren's disease | 1 |
| Horton's disease (giant cell arteritis) | 1 |
| Still's disease of the adult | 1 |
| Willebrand's disease | 1 |
| Thrombotic microangiopathy (HUS, TTP) | 1 |
| Genetic myopathy | 1 |
| Neurofibromatosis type 3 (NF3) or Schwannomatosis | 1 |
| Morton's Nevrome | 1 |
| Otosclerosis | 1 |
| Pheochromocytoma | 1 |
| Retinitis pigmentosa | 1 |
| Retinopathy | 1 |
| Goodpasture's syndrome (vasculitis with anti-MBG) | 1 |
| Guillain-Barre syndrome | 1 |
| Turner syndrome | 1 |
| Attention deficit/hyperactivity disorder (ADHD) | 1 |
| Stomatologic or maxillofacial disorder (SADAM) | 1 |
| Uveitis | 1 |
